# Tenascin C in Lung Diseases

**DOI:** 10.3390/biology12020199

**Published:** 2023-01-28

**Authors:** Chantal Donovan, Xu Bai, Yik Lung Chan, Min Feng, Kin-Fai Ho, Hai Guo, Hui Chen, Brian G. Oliver

**Affiliations:** 1 School of Life Sciences, Faculty of Science, University of Technology Sydney, Sydney, NSW 2007, Australia; 2 Hunter Medical Research Institute and The University of Newcastle, Newcastle, NSW 2050, Australia; 3 Respiratory Cellular and Molecular Biology, Woolcock Institute of Medical Research, Sydney, NSW 2037, Australia; 4 Jockey Club School of Public Health and Primary Care, The Chinese University of Hong Kong, Hong Kong SAR 999077, China; 5 Air Quality Studies, Department of Civil and Environmental Engineering, Hong Kong Polytechnic University, Hong Kong SAR 999077, China

**Keywords:** lung development, foetal programming, particular matter, asthma, COPD, lung cancer

## Abstract

**Simple Summary:**

Tenascin C (TNC) is an extracellular matrix protein involved in numerous cellular processes in development and can be increased in disease; however, its role in lung diseases is less advanced. In this study, we investigated the expression of TNC during lung development, and in the lungs of offspring following maternal particulate matter (PM) exposure, asthma, chronic obstructive pulmonary disease (COPD), and lung cancer. We found that TNC is increased during lung development, in offspring from PM-exposed dams, and in asthma, COPD, and lung cancer tissues. Therefore, targeting TNC may provide a novel therapeutic target for lung diseases.

**Abstract:**

Tenascin C (TNC) is a multifunctional large extracellular matrix protein involved in numerous cellular processes in embryonic development and can be increased in disease, or under conditions of trauma or cell stress in adults. However, the role of TNC in lung diseases remains unclear. In this study, we investigated the expression of TNC during development, in offspring following maternal particulate matter (PM) exposure, asthma, chronic obstructive pulmonary disease (COPD) and lung cancer. *TNC* expression is increased during lung development in biopsy cells, endothelial cells, mesenchymal cells, and epithelial cells. Maternal PM exposure increased TNC and collagen deposition, which was not affected by the removal of PM exposure after pregnancy. *TNC* expression was also increased in basal epithelial cells and fibroblasts in patients with asthma and AT2 and endothelial cells in patients with COPD. Furthermore, there was an increase in the expression of TNC in stage II compared to stage IA lung cancer; however, overall survival analysis showed no correlation between levels of TNC and survival. In conclusion, TNC is increased during lung development, in offspring following maternal PM exposure, and in asthma, COPD, and lung cancer tissues. Therefore, targeting TNC may provide a novel therapeutic target for lung diseases.

## 1. Introduction

The extracellular matrix (ECM) is a diverse group of proteins and glycoproteins, including tenascin C (TNC), collagen, elastin, fibronectin, and proteoglycans, and increased ECM proteins are known to play important roles in the pathogenesis of lung diseases. TNC is a member of the tenascin gene family that is highly expressed during development and is essential for the formation of the central nervous system [1] and bronchial branches in the lung [2]. In adults, the expression of TNC is primarily associated with trauma and/or disease. The complex six-armed structure of TNC enables the interaction of TNC with a wide variety of ligands and receptors (Supplementary Figure S1), including fibronectin for adhesion [3], epidermal growth factor (EGF) receptors [4], α7β1 integrin receptor [5], and α9 integrins [6]. Furthermore, increased expression of TNC has been associated with idiopathic pulmonary fibrosis [7,8,9]; however, the understanding of its role(s) in other lung diseases, such as asthma, chronic obstructive pulmonary disease (COPD) and lung cancer, is less advanced. 

TNC levels are increased in serum from patients with severe asthma compared to patients with mild–moderate asthma [10], and in bronchoalveolar lavage in patients with refractory asthma compared to patients with non-refractory asthma or healthy controls [11]. Interestingly, increased serum TNC levels were associated with improved lung function in patients with severe asthma treated with omalizumab [10]. *TNC* expression in the airway subepithelial reticular basement membrane in patients with asthma is prominently increased after allergen challenge and is a histopathological subepithelial marker to detect disease activity in asthma [12,13]. However, the cellular source(s) of TNC in asthma is unknown. 

In patients with COPD, TNC is increased in the subepithelial layer in large airways and the inner layer in small airways in lung tissue obtained by resection surgery compared to non-smokers [14]. In addition, we have previously shown that TNC is increased in airway smooth muscle cells from COPD smokers compared to non-COPD smokers when stimulated with transforming growth factor (TGF)-β1 [15]. However, the cellular source(s) of TNC in COPD is also unknown.

Increased TNC also promotes tumour progression, epithelial-to-mesenchymal transition, cell growth, migration, and adhesion impairment in breast and prostate cancers [7,16,17,18]. TNC is also increased in lung cancer and the expression of *TNC*, *S100A10*, and *S100A11* can predict survival in patients with lung adenocarcinoma [9]. Furthermore, the overexpression of TNC can promote the spread of lung adenocarcinoma cells in a genetically modified mouse model of lung cancer [9]. However, whether TNC plays a role in other types of lung cancer is unknown.

In this study, we further explored the role of TNC in the lung by assessing TNC during lung development, in the lungs of offspring from pregnant mice exposed to particulate matter (PM), the cellular source(s) of TNC in asthma and COPD, and TNC expression in different stages and subtypes of lung cancer, and network interactions with factors that may play a role in lung cancer development and progression. We hypothesised that TNC would be increased across all of these different disease conditions, irrespective of age, insult, or disease. Importantly, we demonstrate that TNC is increased across a range of different processes, in structural and immune cells, and in disease pathogenesis, and suggest that interactions with integrins may play crucial roles in driving the development and progression of lung diseases. 

## 2. Materials and Methods

### 2.1. Mean TNC Expression during Lung Development Dataset

A total of 81 RNA-seq samples were derived from 12 lungMAP donor subjects from 1 day of life to adult. Data were extracted from Lung-Sorted Cells subsection of the lung gene expression analysis web portal (https://research.cchmc.org/pbge/lunggens/mainportal.html, accessed on 1 Novmber 2022) [19,20,21].

### 2.2. Traffic Related Particulate Matter (PM2.5) Exposure In Vivo

PM was collected as previously described [22] from a busy roadside in Hong Kong by the URG PM samples (URG-2000-30EH, 8 L/min) through a 47 mm Teflon (Pall Life Sciences, Ann Arbor, MI, USA) and (800 °C, 3 h) 47 mm quartz-fibre filters (Whatman, Clifton, NJ, USA).

All animal experiments were approved by the Animal Care and Ethics Committee at The University of Technology Sydney (ETH18-1998). Virgin female BALB/c mice (6 weeks, Animal Resources Centre, Murdoch, WA, Australia) were divided into sham, PM, and pre-exposure groups. The PM group was exposed to PM2.5 (5 μg/day) via intranasal instillation (40 μL) prior to mating for 6 weeks and during gestation and lactation, with sham-exposed mice treated intranasally with saline (40 μL) for the same period. The pre-exposure group was exposed to PM2.5 as above prior to mating and ceased after pregnancy. Lungs were collected from adult offspring and fixed in 10% formalin for tissue staining and analysis. 

### 2.3. Histological Analysis

Lung inflammation and fibrosis were assessed in formalin-fixed paraffin-embedded sections (5 μm) using Hematoxylin and Eosin (H&E) staining, TNC, and Masson’s Trichrome staining. 

*H&E staining*: Lung sections were stained with Mayer’s H&E staining after hydration. Inflammation was assessed with a scoring system double-blinded by two individual investigators. Briefly, the scoring system utilises the following grading criteria (0: no immune cells surrounding the airway; 1: a few immune cells surrounding the airway; 2: a ring of cells comprised of one cell-layer deep; 3: a ring of cells comprised of two cell layers; 4: a ring of cells comprised of three to four cell layers; and 5: a ring of cells comprised of more than four cell layers).

*TNC immunohistochemistry*: A Dako staining kit (Agilent, Sydney, NSW, Australia) was used according to the manufacturers’ instructions. Briefly, the sections were hydrated and underwent heat-induced epitope retrieval by microwaving (Homemaker, Gillman, Australia; EM926ENV; 900 V) for 15 min in citric acid buffer (pH 6.0) followed by cooling in an ice water bath for 15 min. Sections were incubated with primary TNC antibody (1:2000, Sigma-Aldrich, St. Louis, MO, USA) for 40 min, and subsequently incubated with secondary anti-rabbit HRP antibody for 40 min. The sections were then incubated with DAB, and subsequently stained with Mayer’s hematoxylin for nuclear staining. The sections were then dehydrated, and cover slipped for analysis. Two random airway images per mouse were imported into ImageJ, colour channels were split, and the blue channel was converted to a grey scale image. The total area of the grey scale image was measured and the mean grey scale values averaged between the two airways to generate 1 value per mouse.

*Masson’s trichrome staining*: Sections were stained with Trichrome stains according to the manufacturer’s instruction (Sigma-Aldrich, St. Louis, MO, USA). ImageJ was used to determine the % of area stained indicated by blue coloured collagen. 

### 2.4. Asthma Dataset

TNC related data were extracted from different databases. Asthma single cell sequencing data were extracted from Cellular Genetics Informatics Repositories by Teichmann group of Sanger Institute (lung cell atlas, https://asthma.cellgeni.sanger.ac.uk/, accessed on 1 Novmber 2022). TNC data were also sourced from the dataset of lung atlas epithelial, asthma airway epithelial, lung atlas of other cells, asthma atlas of other cells. These data represent the distributions of TNC in different types of airway epithelial and lung cells. 

### 2.5. COPD Dataset

TNC related data were extracted from COPD single cell sequencing data http://www.copdcellatlas.com (accessed on 1 Novmber 2022) [23].

### 2.6. Lung Cancer Dataset

*TNC* expression was also investigated comparing lung cancer and normal lungs. The data were extracted from Gene expression database of Normal and Tumor tissues (GENT) (http://gent2.appex.kr/gent2/, accessed on 1 Novmber 2022) [24]. Overall survival analyses of lung adenocarcinoma (LUAD) and lung squamous cell carcinoma (LUSC) were exported from Gene Expression Profiling Interactive Analysis (GEPIA2) platform (http://gepia2.cancer-pku.cn/#survival, accessed on 1 Novmber 2022) [25], Kaplan–Meier plotter (http://kmplot.com/analysis/, accessed on 1 Novmber 2022), and UALCAN (http://ualcan.path.uab.edu/, accessed on 1 Novmber 2022). *p* values are provided for statistical analysis comparing between healthy individuals and patients with LUAD and LUSC, and between different stages of lung cancer.

### 2.7. Interaction Network Construction

Genomics and proteomics data in GeneMANIA were used to predict TNC gene–gene interactions. The STRING database was used to predict TNC protein–protein interactions. 

### 2.8. Statistical Analysis

Data are expressed as mean or mean ± SEM. For mouse experiments, data are presented from n = 5–8 mice/group. Multiple comparisons were made using one-way ANOVA with Tukey’s post-tests, or the Kruskal–Wallis test with multiple comparisons for nonparametric analyses. * *p* < 0.05, was considered statistically significant. 

## 3. Results

### 3.1. TNC Is Increased during Development and in Offspring from PM2.5-Exposed Dams

**Figure 1:**
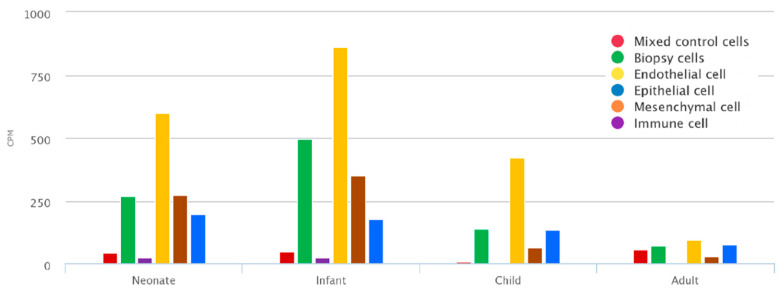
*TNC* is increased during early life lung development. Mean expression of *TNC* at different developmental stages (neonates (up to 30 days), infant (>30 days and <1 year), child (>=1 year and <10 years), and adult (>20 years)) in endothelial cells, epithelial cells, immune cells, mesenchymal cells (defined as CD140+ matrix fibroblasts, myofibroblast/smooth muscle, pericyte, proliferative mesenchymal progenitor, and intermediate fibroblasts), presort stained mixed control and biopsy samples. Data generated from https://research.cchmc.org/pbge/lunggens/mainportal.html (accessed on 1 Novmber 2022) CPM: count per million reads mapped.

To assess the expression of TNC during development, TNC was assessed in human lung tissue, which demonstrates that it is expressed in biopsy cells from neonates (up to 30 days), infants (>30 days and <1 year), and children (>=1 year and <10 years), including endothelial cells, mesenchymal cells (defined as CD140+ matrix fibroblasts, myofibroblast/smooth muscle, pericyte, proliferative mesenchymal progenitor, and intermediate fibroblasts), and epithelial cells (Figure 1). The expression is reduced in adults (>20 years), indicating that *TNC* plays a crucial role in early life lung development. In infants, the expression is highest in mesenchymal cells, followed by epithelial cells (Figure 1). 

**Figure 2:**
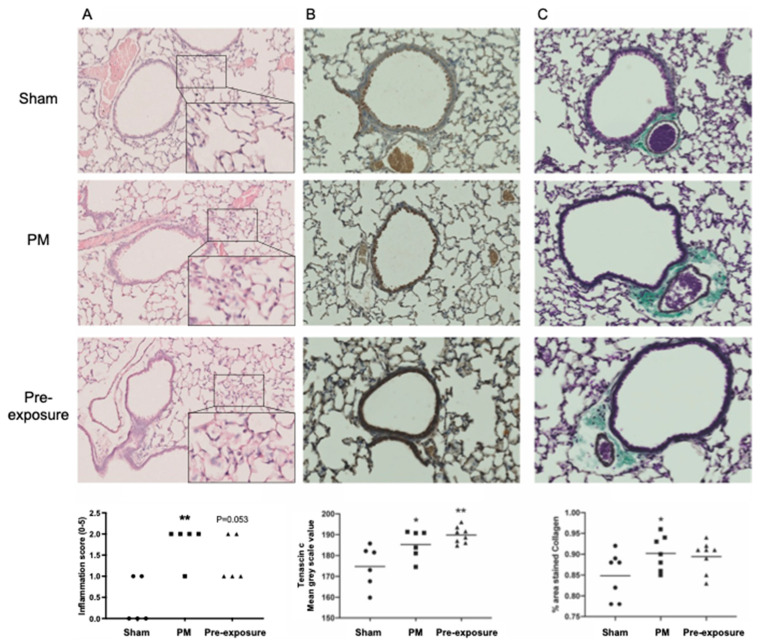
TNC is increased in offspring from PM-exposed dams. (**A**–**C**) Representative images of lung sections in male offspring at 13 weeks. (**A**) Hematoxylin and eosin staining and analysis, n = 5. (**B**) TNC immunohistochemistry and analysis, n = 6–8. (**C**) Masson’s Trichrome staining and analysis, n = 7–8. * *p* < 0.05, ** *p* < 0.01 vs. sham. PM: particulate matter.

Given that TNC was increased during lung development, we next aimed to assess whether an insult in the mothers (maternal PM exposure) would also lead to increased TNC responses in offspring, and whether this response is associated with inflammation and lung remodelling. Maternal PM exposure increased inflammation in offspring compared to those from sham-treated dams (*p* < 0.01; Figure 2A). The removal of maternal PM exposure after pregnancy (pre-exposure) did not reduce inflammation in offspring compared with offspring from dams continuously exposed to PM during and after pregnancy (*p* = 0.053 pre-exposure vs. sham; Figure 2A). TNC staining was ubiquitous throughout the lung and increased in the PM and pre-exposure groups compared to the sham (*p* < 0.05 PM vs. sham; *p* < 0.01 pre-exposure vs. sham; Figure 2B). TNC is also known to drive fibrosis and increase collagen expression [26]; thus, we next assessed collagen deposition using Masson’s trichrome staining. Collagen staining was increased in the offspring from PM-exposed dams compared to those from sham-treated dams (*p* < 0.05 PM vs. sham), which was not significantly mitigated by the removal of PM after pregnancy in the dams (Figure 2C). Together, these data suggest that TNC is increased during lung development in structural cells and that maternal PM exposure results in an increase in TNC expression in offspring that is sustained even if PM exposure is ceased after pregnancy. These data also suggest that changes in TNC levels during foetal development/early life may result in increased TNC levels in the lung in later life.

### 3.2. TNC Expression Is Higher in Basal Epithelial Cells Compared to Parenchyma in Healthy Airways, and in Asthmatic Samples Compared to Healthy Controls

**Figure 3:**
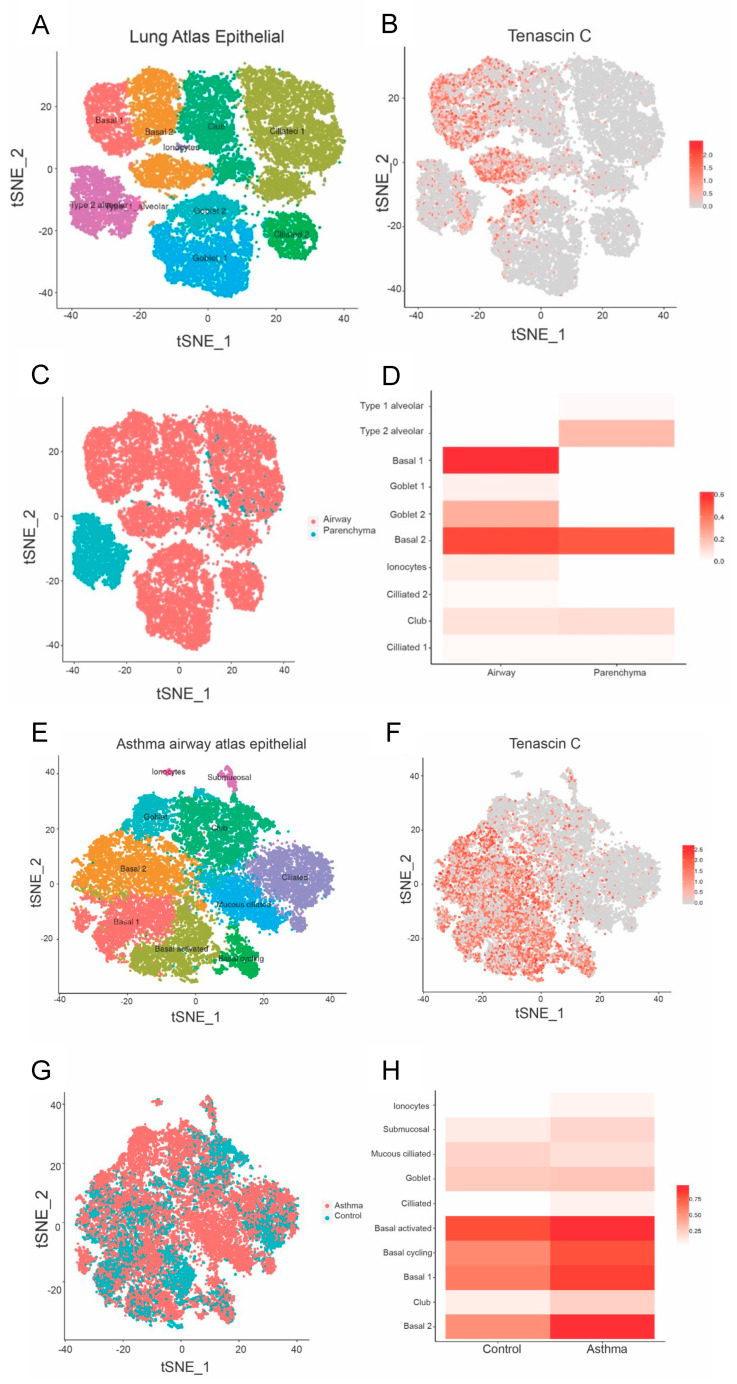
*TNC* expression is increased in basal epithelial cells compared to parenchyma in healthy airways, and in asthmatic samples compared to healthy controls. (**A**–**D**) Single-cell RNA sequencing clustering analysis in healthy samples generated from https://asthma.cellgeni.sanger.ac.uk/ (accessed on 1 Novmber 2022). (**A**) Data from the epithelial lung atlas showing lung cell distribution. (**B**) *TNC* distribution in different cells corresponding to lung atlas. (**C**) Type of lung cell distribution in airway and parenchyma. (**D**) *TNC* distribution in airway and parenchyma correlating to different cell types. (**E**–**G**) Single-cell RNA sequencing clustering analysis in asthma airway. (**E**) Asthma airway atlas epithelial showing abundance of different types of lung cell distribution in asthmatic patients. (**F**) *TNC* distribution in different epithelial cells in asthmatic patients. (**G**) Epithelial cell distribution in asthmatic and control patients. (**H**) *TNC* distribution in control and asthmatic patients.

**Figure 4:**
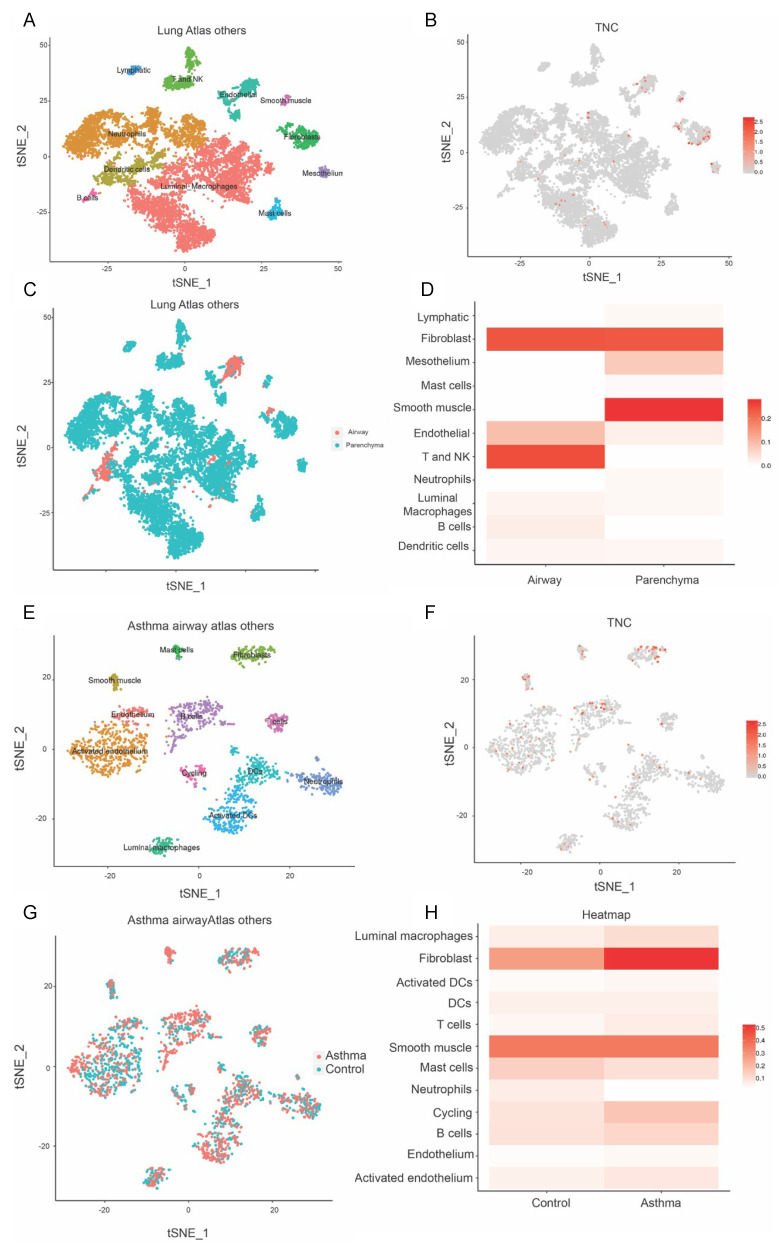
*TNC* expression is increased in fibroblasts in asthmatic samples compared to healthy controls. (**A**–**D**) Single-cell RNA sequencing clustering analysis in healthy samples generated from https://asthma.cellgeni.sanger.ac.uk/ (accessed on 1 Novmber 2022). (**A**) Data from the lung atlas of other cells showing cell distribution. (**B**) *TNC* distribution in different cells corresponding to lung atlas. (**C**) Type of lung cell distribution in airway and parenchyma. (**D**) *TNC* distribution in airway and parenchyma correlating to different cell types. (**E**–**G**) Single-cell RNA sequencing clustering analysis in asthma airway. (**E**) Asthma atlas of other cell types showing the abundance of different lung cells distribution in asthmatic patients. (**F**) *TNC* distribution in different cells in asthmatic patients. (**G**) Cell distribution in asthmatic and control patients. (**H**) *TNC* distribution in other cells in control and asthmatic patients.

Asthma is associated with environmental insults in early life, predisposing to allergic airway hyperresponsiveness and disease outcomes later in life. Given that TNC was found to be increased during early life lung development and in offspring from PM-exposed dams, we next aimed to assess *TNC* expression in patients with asthma compared to healthy controls. Furthermore, as TNC was ubiquitously expressed in the lungs of offspring at 13 weeks of age, we aimed to further define the cellular source of TNC; therefore, we extracted single-cell RNA sequencing data from the Lung Cell Atlas. *TNC* is distributed in basal cells and goblet cells (Figure 3A–D). In asthmatic patients, *TNC* is found in activated cycling basal cells and club cells (Figure 3E–H). *TNC* expression is also higher in basal cells in patients with asthma compared to controls. Furthermore, *TNC* is expressed in fibroblasts, airway smooth muscle, T cells, and NK cells (Figure 4A–D). In asthmatic patients, *TNC* levels are higher in fibroblasts, B cells, and endothelium compared to controls (Figure 4E,F). Together, these data suggest that *TNC* can be expressed by a variety of cell types and that expression of *TNC* is increased in structural (fibroblast, airway smooth muscle, and endothelium) and immune (T cells and NK cells) cells in patients with asthma.

### 3.3. TNC Expression Is Increased in AT2A Cells and Endothelial Cells (Peribronchial Vascular Endothelial Cells) in COPD Compared to Controls

**Figure 5:**
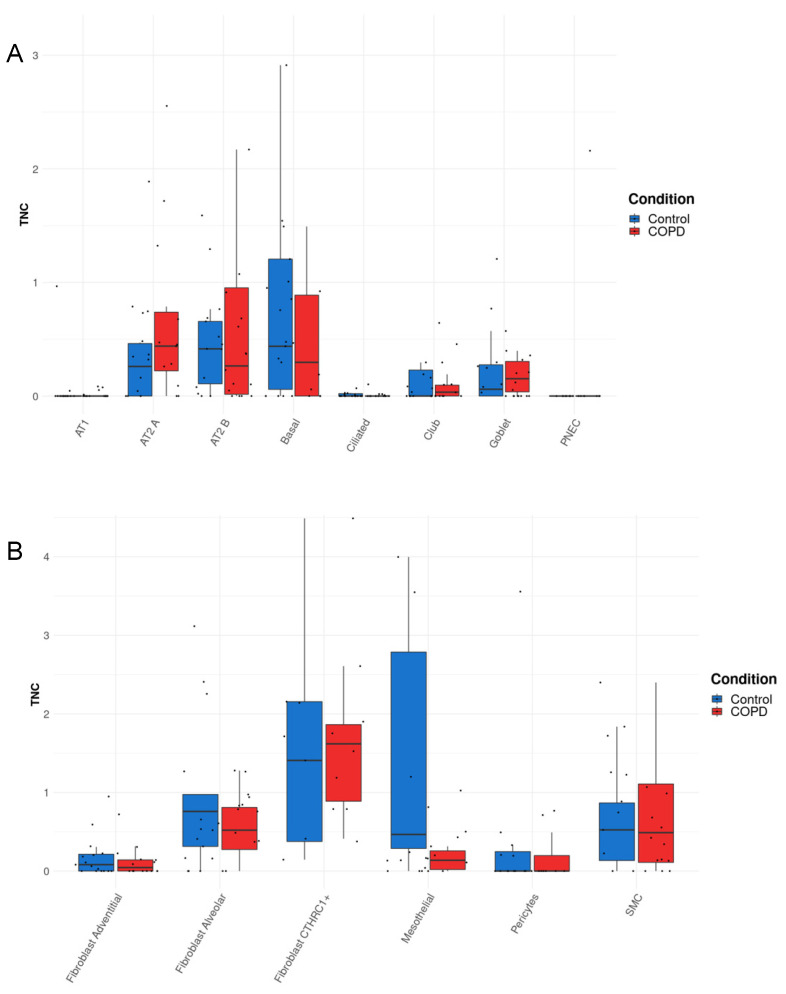

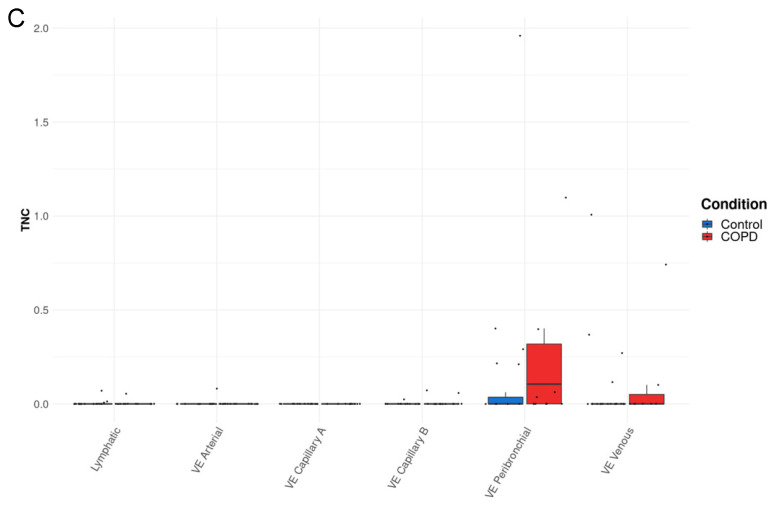
*TNC* expression is increased in AT2A cells and endothelial cells (peribronchial vascular endothelial cells) in COPD compared to control lungs. (**A**–**C**) TNC expression in parenchymal tissue explanted from the lung of patients with COPD or control donors generated from http://www.copdcellatlas.com (accessed on 1 Novmber 2022) [23].

Our results from Figure 1, Figure 2, Figure 3 and Figure 4 demonstrated key roles for TNC during lung development, in offspring born to PM-exposed dams, and in asthma, which commonly starts in childhood. Thus, to determine whether TNC levels were also altered in lung diseases that develop later in life, we next sought to assess the cellular source(s) of *TNC* in COPD and extracted single-cell RNA sequencing data from the COPD Cell Atlas [23]. *TNC* is distributed in the epithelial, stromal, and endothelial cells in parenchymal samples from both control and COPD lungs (Figure 5A–C). In patients with COPD, *TNC* was increased in AT2A cells (defined as expressing *SFTPC, ERBB4, TNIK, TCF12, FOXP1, STAT3, YAP*, and *TEAD1* [23]) and peribronchial vascular endothelial cells compared to control lungs (Figure 5A,C). In contrast, TNC in AT2B cells (defined as expressing *SFTPC, SFTPA1, SFTPA2,* and *ETV5*) appeared to be reduced in patients with COPD compared to controls (Figure 5A). These data, together, suggest that *TNC* is increased during development and in disease states irrespective of the age of disease onset.

### 3.4. TNC Expression Is Increased in Lung Cancer but Does Not Correlate with Lung Cancer Survival

**Figure 6:**
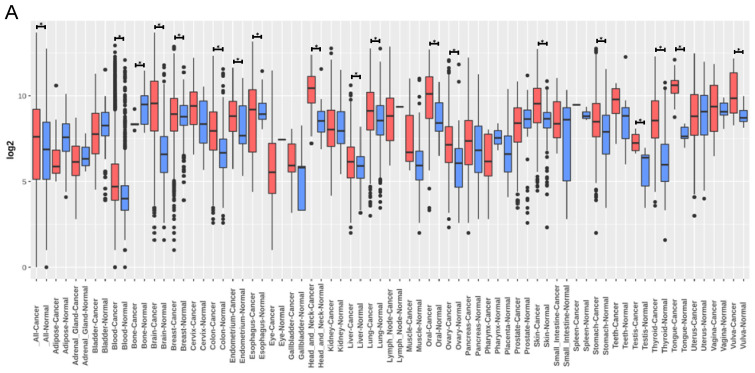

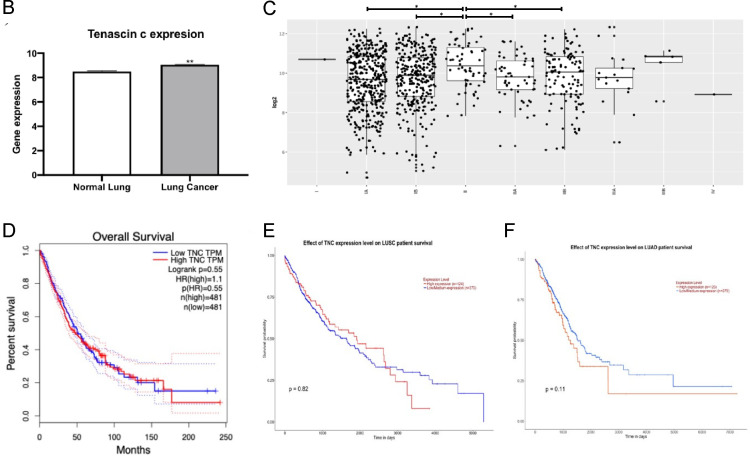
*TNC* expression is increased in lung cancer, but does not correlate with lung cancer survival. (**A**–**C**) *TNC* expression generated from http://gent2.appex.kr/gent2/ (accessed on 1 Novmber 2022) [24]. (**A**) *TNC* expression in different tissues. (**B**) TNC expression in normal lung (n = 508) and lung cancer (n = 2362). (**C**) *TNC* expression in different subtypes of lung cancer (I, IA, IB, II, IIA, IIB, IIIA, IIIB, IV). (**D**) Overall survival curve of the high and low expression of *TNC* in patients with lung cancer (generated from http://gepia2.cancer-pku.cn/#survival, accessed on 1 Novmber 2022) [25]. (**E**,**F**) Overall survival curve generated using Kaplan–Meier plotter (http://kmplot.com/analysis/, accessed on 1 Novmber 2022) and UALCAN (http://ualcan.path.uab.edu/, accessed on 1 Novmber 2022) (**E**) Overall survival curve of the high and low expression of *TNC* in patients with lung squamous cell carcinoma (LUSC). (**F**) Overall survival curve of the high and low expression of *TNC* in patients with lung adenocarcinoma (LUAD). * *p* < 0.05; ** *p* < 0.01 compared to normal lung. HR: hazards ratio; TPM: transcripts per million.

**Figure 7:**
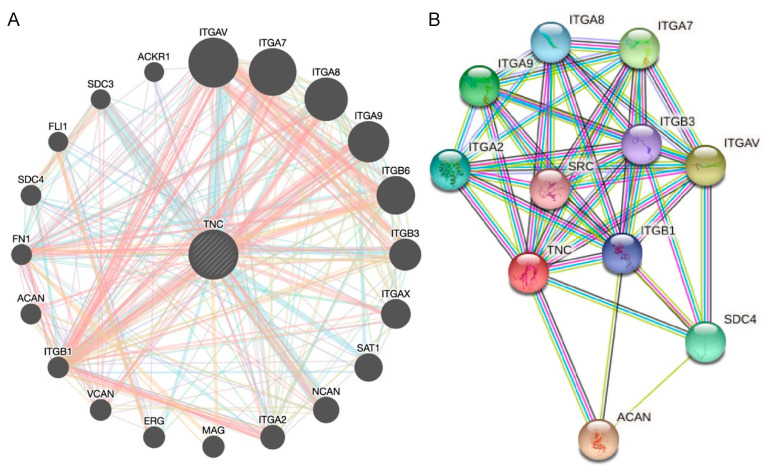
Analysis of neighbouring gene networks in lung cancer. (**A**) The gene–gene interaction network of TNC constructed using GeneMania. (**B**) The protein–protein interaction network of TNC generated using STRING.

We next sought to assess the cellular source(s) of TNC in lung cancer, which is another adult-onset lung disease. Publicly available data from GENT2 provide the comparison of gene expression patterns between normal and cancerous tissues (Figure 6A). GENT2 provides gene expression from more than 68,000 samples generated by the Affymetrix U133A or U133Plus2 microarray platforms. A search of “TNC” provides data on gene expression in both normal and lung cancer samples. *TNC* expression was increased in lung cancer tissue compared to normal lung tissue (*p* < 0.05; Figure 6B). GENT2 data also showed *TNC* expression levels at different stages of lung cancer. There is a higher level of *TNC* in Stage II compared to Stage IA with no differences between later stages of lung cancer (Figure 6C). TNC is currently not considered as a prognostic marker for cancer, but this could be a result of the lack of human lung samples studied. Overall survival analysis shows no correlation between levels of *TNC* and lung cancer (Figure 6D). Further segregation of lung cancer data into lung adenocarcinoma and lung squamous cell carcinoma shows the same result (Figure 6E,F). In lung adenocarcinoma and lung squamous cell carcinoma, the differences in the 10-year survival rate are not significant. Gene–gene interaction and protein–protein interaction analyses of TNC in lung cancer showed that TNC plays a multifunctional role in the ECM and inflammation (Figure 7A,B, Table 1). These data suggest that TNC and its interactions with ECM and inflammatory proteins may be important in the development and progression of lung cancers. 

## 4. Discussion

TNC is an important matricellular ECM protein involved in development and morphogenetic events such as inflammation, injury repair or cancer. Whilst some studies have assessed its expression in the lung, there remain gaps in our knowledge. In this study, we explored TNC expression during lung development, in the lungs of offspring with maternal PM2.5 exposure, the cellular source of *TNC* in asthma and COPD lungs, and in different stages and subtypes of lung cancers. We demonstrate that TNC is increased across a range of different physiological and pathophysiological processes, in both structural and immune cells, which suggests that TNC interactions with integrins may drive the lung development and progression of lung diseases.

Here, we show that TNC is increased in structural cells during lung development which is consistent with other studies showing that expression of *TNC* in the lungs peaks during alveolarisation [27], and confirm previous studies of reduced/limited expression of *TNC* in adults [28]. Previous studies have shown that there was a 36% reduction in airway branches in *TNC*-deficient mice at embryonic day 12.5 compared to wild type mice and that this reduction in branches also affected alveoli size at postnatal day 2 in the *TNC*-deficient mice [29]. We have previously shown that chronic maternal exposure to PM during pregnancy results in increased lung dysfunction in male offspring [22]. These offspring have increased airway inflammation (macrophages, neutrophils, and eosinophils), increased mitochondria density and reactive oxygen species levels, and a trend towards increased epithelial thickness and mucous secreting cells. Using this model, we now demonstrate that these male offspring also have increased tissue inflammation, collagen and TNC levels compared to those from sham-treated dams. Importantly, PM2.5 pre-exposure does not affect inflammation or TNC levels in male offspring, suggesting that maternal PM2.5 exposure may cause epigenetic modifications to TNC that are passed on to the offspring. Notably, in the heart, TNC can be demethylated by angiotensin-II [30], which suggests that factors that may increase TNC demethylation by maternal PM exposure could also alter TNC expression in offspring. Together, these studies suggest that increased TNC may be associated with the inflammatory response and/or profibrotic changes that will remain as permanent structural modification of the lung. Future studies assessing the cellular sources of TNC, TNC methylation, and correlations between inflammatory and pro-fibrotic mediators and TNC in offspring from dams exposed to PM2.5 are warranted.

In utero exposures, genetic factors, environmental stimuli and/or maternal stress can predispose asthma development. Maternal exposure to PM2.5 was found to increase the risk of asthma in children [31], and we showed that chronic maternal PM exposure results in a greater magnitude of airway hyperresponsiveness and eosinophilic airway inflammation during experimental asthma when compared to offspring from sham-exposed dams [22]. However, no studies to date have examined the role of TNC responses in translating these maternal PM exposure-induced effects in offspring and these are warranted in future studies. Furthermore, given that maternal PM exposure increases the severity of these lung sequelae in offspring, we sought to examine TNC responses in the development of chronic lung diseases, including asthma, COPD, and lung cancer.

*TNC* expression is increased in a variety of cell types, including structural (fibroblast, airway smooth muscle, and endothelium) and immune (T and NK) cells in patients with asthma compared to healthy controls. However, there is no definitive evidence that asthma-associated TNC enrichment in these cell types precipitates disease features associated with asthma, including inflammation, airway remodelling, and altered lung function. Interestingly, one study showed that increased serum TNC responses are linked with improvements in lung function in severe asthma [10]; however, whether serum TNC is reflective of lung TNC levels is yet to be determined. In a separate study, *TNC* knockout mice have attenuated allergic inflammation and reduced eosinophil infiltration, which was associated with reduced expression of type 2 cytokines, IL-5, and IL-13 [32].

Deposition of TNC is also associated with IL-5 release, which promotes eosinophil maturation. Atopic asthma patients with increased TNC basement membrane thickness were also found to have higher eosinophils, T-lymphocytes, macrophages, IL-4^+^ cells [33], and anti-IL-5 treatment reduced deposition of TNC in the bronchial subepithelial basement membrane of mild atopic asthmatics [12]. Furthermore, TNC has also been shown to activate MAPK signalling through β1 and β3 integrins, which in turn upregulates matrix metalloproteinase-1 and mediates airway remodelling in asthma [34]. Bronchial biopsies from asthmatic patients were also found to have higher fibrin levels along with TNC accumulation [35]. However, whether these pathways are activated in fibroblasts, the airway smooth muscle, and endothelial, T, and NK cells remains to be elucidated. However, we propose that structural (e.g., fibroblast or epithelial), cell-derived TNC responses may be more accurately correlated with inflammation, remodelling, and disease severity in patients with asthma compared to serum TNC, and this avenue should be explored in future studies.

We recently showed that airway smooth muscle cells from patients with COPD exhibit robust and elevated TNC responses following stimulation with TGF-β1 [15] and, in the current study, show that increased *TNC* responses also occur in AT2 and endothelial cells in COPD. COPD-associated TNC responses have also been linked with concomitant increases in the expression of many ECM-associated factors, including collagen species, elastin fibres, collagens, versican, and fibronectin [14]. Collectively, these data suggest that TNC from multiple lung cell sources may orchestrate pro-fibrotic responses and drive remodelling events in the lung during COPD pathogenesis, which warrants further examination of its functional roles and potential for therapeutic targeting in this context.

TNC is a metastasis promoter that is widely studied in cancer and is present in non-small cell lung cancer (NSCLC), which accounts for 80–85% of all lung cancers [36]. TNC can increase lung cancer metastasis through blood vessel invasion [37]. In humans, *TNC* gene expression is also eighteen times higher in recurring NSCLC compared to non-recurrent NSCLC patients [36]. In our analysis, *TNC* was increased in early stage lung cancer (Stage II) compared to Stage IA, with no differences evident between later stages of lung cancer, and overall survival did not correlate with TNC levels, irrespective of the type of lung cancer (adenocarcinoma or squamous cell). This finding is in contrast to a previous study which showed that TNC could predict survival in lung adenocarcinoma patients [9]. These discrepancies may be explained by differences in quartiles used to assign patient subgroups, dataset size, stage of disease, age, gender, smoking history, or mutational load, and highlight the heterogeneity of patients with lung adenocarcinoma. Nevertheless, increased TNC is present in lung cancer patients, and gene and protein interaction analysis showed that TNC interacts with ECM and inflammatory proteins, which warrants further investigation into their roles in the development and progression of lung cancer.

## 5. Conclusions

TNC plays an important role during lung development, in offspring following maternal PM2.5 exposure, in asthma and COPD, and in lung cancer. Identifying novel therapeutic drugs that target TNC or pathways that TNC can regulate may provide novel treatment avenues for lung diseases.

## Figures and Tables

**Table 1:** Kyoto Encyclopedia of Genes and Genomes (KEGG) enrichment and GO analysis of neighbouring genes for TNC.

	Term ID	Term Description	Observed Gene Count	Background Gene Count	Strength	False Discovery Rate
KEGG	hsa04512	ECM-receptor interaction	13	88	2.14	5.40 × 10^−23^
	hsa04510	Focal adhesion	13	198	1.79	3.94 × 10^−19^
	hsa05412	Arrhythmogenic right ventricular cardiomyopathy	11	76	2.13	3.94 × 10^−19^
	hsa05410	Hypertrophic cardiomyopathy	10	89	2.02	1.93 × 10^−16^
	hsa05414	Dilated cardiomyopathy	10	95	1.99	2.85 × 10^−16^
Biological Process	GO:0007229	Integrin-mediated signalling pathway	11	94	2.04	2.66 × 10^−16^
	GO:0030198	Extracellular matrix organization	14	338	1.59	2.66 × 10^−16^
	GO:0007155	Cell adhesion	16	925	1.21	3.71 × 10^−14^
	GO:0031589	Cell-substrate adhesion	11	182	1.75	4.42 × 10^−14^
	GO:0007160	Cell-matrix adhesion	10	127	1.87	1.22 × 10^−13^
Molecular Function	GO:0050840	Extracellular matrix binding	8	56	2.12	5.26 × 10^−12^
	GO:0005178	Integrin binding	9	147	1.76	4.48 × 10^−11^
	GO:0050839	Cell adhesion molecule binding	11	538	1.28	2.24 × 10^−9^
	GO:0001968	Fibronectin binding	5	27	2.24	1.36 × 10^−7^
	GO:0005102	Signalling receptor binding	12	1581	0.85	7.11 × 10^−6^
Cellular Component	GO:0008305	Integrin complex	10	30	2.49	2.19 × 10^−19^
	GO:0005925	Focal adhesion	15	405	1.54	1.99 × 10^−18^
	GO:0098797	Plasma membrane protein complex	15	547	1.41	9.63 × 10^−17^
	GO:0070161	Anchoring junction	16	820	1.26	4.98 × 10^−16^
	GO:0098802	Plasma membrane signalling receptor complex	11	169	1.78	2.29 × 10^−15^

## Data Availability

Lung development data were extracted from https://research.cchmc.org/pbge/lunggens/mainportal.html (accessed on 1 Novmber 2022) [19,20,21]. Asthma data were extracted from Cellular Genetics Informatics Repositories by Teichmann group of Sanger Institute (lung cell atlas, https://asthma.cellgeni.sanger.ac.uk/) (accessed on 1 Novmber 2022). COPD data were extracted from www.COPDcellatlas.com (accessed on 1 Novmber 2022) [23]. Lung cancer data were extracted from Gene expression database of Normal and Tumor tissues (GENT) (http://gent2.appex.kr/gent2/, accessed on 1 Novmber 2022) [24]. Overall survival analysis of lung adenocarcinoma (LUAD) and lung squamous cell carcinoma (LUSC) were exported from Gene Expression Profiling Interactive Analysis (GEPIA2) platform (http://gepia2.cancer-pku.cn/#survival, accessed on 1 Novmber 2022) [25], Kaplan–Meier plotter (http://kmplot.com/analysis/, accessed on 1 Novmber 2022) and UALCAN (http://ualcan.path.uab.edu/) (accessed on 1 Novmber 2022).

## Supplementary Materials

The following supporting information can be downloaded at: https://www.mdpi.com/article/10.3390/biology12020199/s1, Figure S1. (A) Simplified structure of TNC. TNC has six arms that are each comprised by region of oligo assembly, EGF-like domain, Constant FNIII domains, Variable FNIII domains and fibronectin globe. Epidermal growth factor (EGF); Fibronectin (FN). Multiple functions of TNC. (B) TNC crosslinks with FIII domains. (C) TNC binds to the cell membrane through integrin. (D) Fibronectin globe binds to TLR4. (E) EGF L binds to EGFR in fibroblast. (Created with Biorender.com).
